# Dissipation of Three Fungicides and Their Effects on Anthocyanins and Color of Monastrell Red Wines

**DOI:** 10.3390/ijms20061447

**Published:** 2019-03-22

**Authors:** Noelia Briz-Cid, Raquel Rial-Otero, Miguel A. Cámara, José Oliva, Jesus Simal-Gandara

**Affiliations:** 1 Nutrition and Bromatology Group, Department of Analytical and Food Chemistry, CITACA, Faculty of Food Science and Technology, University of Vigo, Ourense Campus, E32004 Ourense, Spain; nbriz@uvigo.es (N.B.-C.); raquelrial@uvigo.es (R.R.-O.); 2 Department of Agricultural Chemistry, Geology and Pedology, Faculty of Chemistry, University of Murcia, Campus de Espinardo, 30100 Murcia, Spain; mcamara@um.es

**Keywords:** fungicides, wine quality, winemaking process, anthocyanins, antioxidant activity

## Abstract

The effect of fungicides on fermentation is of paramount importance to control the quality and safety of wines. In this work, the quality (enological parameters, color, phenolic content, antioxidant activity, and fungicide residues) of wines from Monastrell grapes fortified with iprovalicarb, mepanipyrim, and tetraconazole fungicides was evaluated. Along the winemaking process, initial residues of mepanipyrim and tetraconazole were removed in more than 90% while the dissipation of iprovalicarb was around 73%. Significant statistical differences were found in the presence of iprovalicarb and mepanipyrim residues, especially at the highest concentration assayed. For both fungicides, increases in the volatile acidity (between 4 and 8.6 times), the lactic acid content (between 8.6 and 20.5 times), the percentage of polymeric anthocyanins (between 1.3 and 1.7 times), and also a slight increase of the total phenolic index and the total anthocyanin content determined by spectrophotometry were observed. On the contrary, the total monomeric anthocyanins content decreased about 16.3% and 28.6% in the presence of iprovalicarb and mepanipyrim, respectively. These results could be related to a higher development of acetic acid or lactic bacteria in the presence of these fungicides. The color of the final wines was also different in comparison with the control, with a higher yellow component, color intensity, tonality, and hue angle because of pH changes in the medium. Tetraconazole fermentations had a more similar trend to the control wine, probably due to the lower concentration of this fungicide in the grape must at the initial time. No effects on the antioxidant activity was observed for any of the target fungicides. A multivariate statistical analysis was done to view the interrelationships between different variables (color and anthocyanins profile). The obtained model allowed the wines to be separated according to the fungicide treatment applied.

## 1. Introduction

The control of vine diseases is one of the most important factors to obtain quality grapes and, consequently, quality wines. Fungicide treatment during the season is the most effective method to fight against them. Iprovalicarb, mepanipyrim, and tetraconazole are fungicides frequently applied on vineyards to combat different fungal diseases. They belong to different chemical groups and have different modes of action. Iprovalicarb is a valinamide carbamate fungicide used against *Plasmopara viticola* in viticulture practices, and its target site of action is the cellulose synthase of the cell wall. Mepanipyrim is an aniline–pyrimidine fungicide used against *Botrytis cinerea* and affects methionine biosynthesis. Tetraconazole is used to fight against *Uncinula necator* in grapevines. It belongs to the triazoles chemical group and acts to avoid sterol biosynthesis on the membrane [1].

The concentration of fungicide residues in grapes depends on the active substance, formulation, applied dose, time interval from application, and climatological conditions [2]. Diverse studies have found residues of fungicides at trace levels on vinification grapes after harvest [3,4,5], although levels in the range of 0.5–2.5 mg·kg^−1^ were reported in others [6,7,8]. Some enotechnological processes (crushing, pressing, racking, clarification, and filtration) can influence the dissipation of fungicides along the winemaking process, reducing their levels [4,6,9,10,11].

The color of red wines is a quality factor highly affected by a different distribution of monomeric and polymeric anthocyanins [12,13]. Phenolic compounds are considered the origin of color, taste, and astringency (tannins) and have a nutritional interest due to their antioxidant properties [14,15]. The presence of fungicide residues on grape must may modify the color and the phenolic composition of wines [16,17,18,19,20]. Mulero and coworkers observed that the addition of different fungicides (famoxadone, fenhexamid, fluquinconazole, kresoxim-methyl, quinoxyfen, and trifloxystrobin) to Monastrell grapes led to wines with different phenolic profiles [16]. Modifications on the phenolic content of Tempranillo and Graciano wines were observed when grapes were treated with metrafenone and boscalid + kresoxim-methyl under good agricultural practices [17,18] and fenhexamid and mepanipyrim under critical agricultural practices [18,19]—monomeric anthocyanins being the most affected compounds. The addition of a tetraconazole commercial formulation to Mencía grapes reduced the phenolic content of wines by more than 45% [20]. Anthocyanins (reductions of about 64–75%) and flavan-3-ol monomers (reductions of about 35%) were the most affected compounds. Published results suggest that the effects depend on the cultivar, the kind of fungicide and its concentration, and the considered phenolic family.

In the present study, for the first time, the effect of three fungicides (iprovalicarb, mepanipyrim, and tetraconazole), added to the grape must at different concentration levels, on the chromatic characteristics (color and phenolic composition) of Monastrell red wines of the Designation of Origin (DO) Jumilla (southeast Spain) was evaluated. Monastrell grapes are the most representative of DO Jumilla, growing on over 80% of the cultivated area [21]. The dissipation of the fungicides during the winemaking process was studied, as well as the effect of the fungicides on several enological parameters and antioxidant activity.

## 2. Results and Discussion

### 2.1. Fungicide Dissipation

Dissipation with winemaking depended on the target fungicide (Table 1)—97% (in mass units) for mepanipyrim, 91–92% for tetraconazole, and 72–74% for iprovalicarb. Since the Maxima Residue Level (MRL) for tetraconazole in grapes is four times lower than for the other two fungicides, their residual levels in clarified wines decreased as follows: 2.6–6.0 mg·L^−1^ (iprovalicarb) > 0.2–0.7 mg·L^−1^ (mepanipyrim) > 0.2–0.5 mg·L^−1^ (tetraconazole).

Between 33–38% (in mass units) of the residues of iprovalicarb and mepanipyrim and 65–72% of tetraconazole were eliminated in the grape pomace after pressing. Cabras et al. [9] have already reported a high affinity of tetraconazole for the solid matter, whereas an inverse behavior was found for iprovalicarb [3]. An additional 2–6% was removed with the lees, whereas the clarification step had an important impact on the mepanipyrim residues (a decrease of 22–24% with respect to the wine before clarification).

For mepanipyrim, the sum of the amounts (mg) in the must-wine after pressing and the grape pomace was lower than the initial amount in crushed grapes. The aqueous degradation of mepanipyrim in aqueous solutions [22,23] and in a grape juice analog [24] could be the explanation for this finding.

Taking into account that the acceptable daily intake (ADI) for iprovalicarb, mepanipyrim, and tetraconazole is 0.015, 0.012, and 0.004 mg·kg^−1^ bw per day, respectively [25,26,27], in the case of a reference person weighing 70 kg, the maximum allowable intake would be 1.05, 0.84, and 0.28 mg·day^−1^. Thus, a moderate consumption of wine (a cup of 150 mL) represents an intake lower than 25% of the ADI for the most dissipated fungicides (mepanipyrim and tetraconazole) and 37.14% and 85.57% of the ADI for iprovalicarb at 2MRL and 5MRL, respectively (Table 1).

### 2.2. Enological Parameters

With the evolution of must density during fermentation, it was proved that all vinifications had a regular course. Therefore, the initial fungicide levels in grapes do not inhibit yeast metabolism. The global parameters obtained for wines in the presence and absence of fungicides are shown in Table 2. Statistically significant differences were found in most of the enological parameters in the presence of fungicides, especially with iprovalicarb and mepanipyrim residues at the highest concentration assayed (5MRL). The volatile acidity of these wines (expressed as acetic acid concentration) was 8.6 times higher than that observed in the control wine. High acetic acid values in wines are considered a fault and are typically associated with the respiratory metabolism of ethanol by acetic acid bacteria [28]. However, the effect of lactic acid bacteria should be also taken into account. Lactic acid bacteria can metabolize malic acid, sugars, and citric acid to produce lactic acid. However, the development of heterofermentative bacteria, such as those from the genus *Leuconostoc* and *Oenococcus*, produce carbon dioxide, ethanol, and acetic acid in addition to lactic acid [29]. It is hypothesized that a secondary malolactic fermentation happened spontaneously in the presence of fungicide residues, especially with iprovalicarb and mepanipyrim. In fact, the extension of the malolactic fermentation was fungicide concentration dependent and did not occur in the control samples (without fungicides). Consequently, a de-acidification of high acid wines, by the transformation of malic acid into lactic acid, was observed [30]. Although, in general, bacterial development occurs after yeast development, the presence of fungicide residues could have helped the development of bacteria by extending the lag phase of the yeast. Under these conditions, with high sugar levels in the medium, an important increment of the volatile acidity during the malolactic fermentation could be observed [29].

### 2.3. Phenolic Characterization

Iprovalicarb and mepanipyrim produced significant increments on the total phenolic index and the total anthocyanin content measured by spectrophotometry, these increments being fungicide concentration dependent (Table S1 of Supplementary Material and Table 2). For both fungicides, a different distribution of the monomeric and polymeric anthocyanins was obtained. The monomeric anthocyanin content decreased about 1.5–3.8 times with respect to the control wine while the content of the polymeric forms was 1.3–1.6 times higher (Table S1 of Supplementary Material). For tetraconazole at 5MRL, a similar trend was observed although in a lower extension.

**Figure 1:**
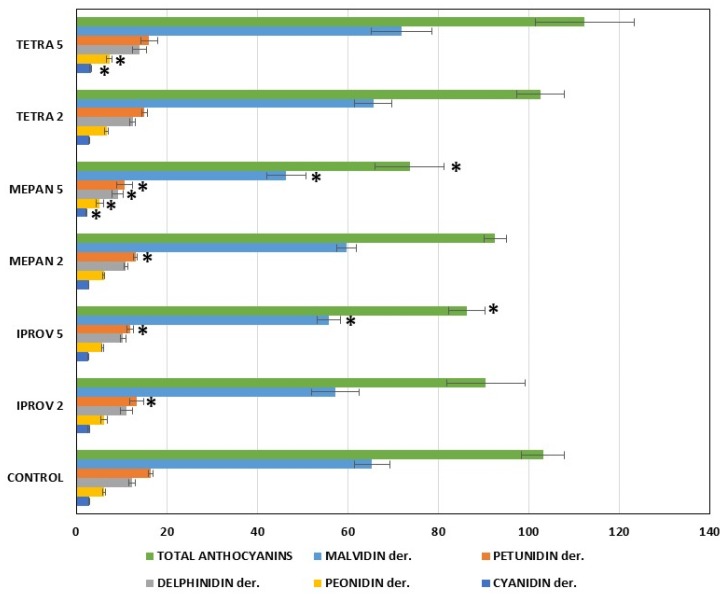
Results correspond to the average ± standard deviation (*n* = 3) of the anthocyanin concentration (mg·L^−1^) extracted from the control and treated wines. * Statistically significant differences at *p* < 0.05.

In addition, significant differences were also found in the total monomeric anthocyanin content determined by High Performance Liquid Chromatography (HPLC) in the presence of iprovalicarb and mepanipyrim residues at 5MRL (Table S1 of Supplementary Material). Decreases of about 16.3% and 28.6%, in the total monomeric anthocyanin content with respect to the control wine were observed for iprovalicarb and mepanipyrim, respectively. The most affected anthocyanins were the malvidin (decreases between 14.6% and 29.0%) and petunidin (decreases between 28.1% and 35.5%) derivatives. No significant differences were found for the other fungicide treatments (Figure 1). Differences in the anthocyanin content between the spectrophotometric and the chromatographic methods were observed, because only free anthocyanins are determined by HPLC while the contribution of other pigments is possible by spectrophotometry [31]. Since vinifications were done at the same time, and in the same way, and also compared with a control vinification (without fungicides), the observed behavior should be attributed only to the presence of fungicide residues in the must.

Similar results were found in Tempranillo and Graciano wines treated with mepanipyrim, with decreases in the total monomeric anthocyanin content of 24.7–36.4% [19]. Low monomeric anthocyanin concentrations in wines in the presence of other fungicides (metrafenone, boscalid kresoxim-methyl, famoxadone, and trifloxystrobin) were also reported [16,17].

The presence of acetic acid or lactic acid bacteria could explain the higher percentage of polymeric anthocyanins and the decrease of monomeric anthocyanins (especially malvidin derivatives) in the presence of iprovalicarb and mepanipyrim at 5MRL. Acetic acid bacteria can oxidize ethanol to acetaldehyde [32,33]. The presence of acetaldehyde was associated with a rapid polymerization process between anthocyanins and catechin or tannins, increasing wine color stability [30,34]. In fact, the condensation of malvidin-3-*O*-glucoside with acetaldehyde was demonstrated in model solutions [35].

### 2.4. Color Changes

**Figure 2:**
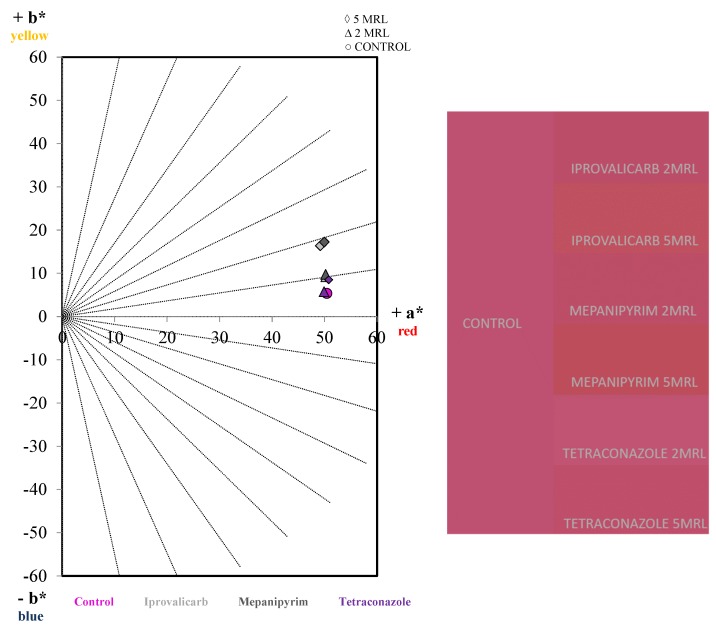
CIELab space for the control and treated wines (**left**), showing how iprovalicarb and mepanipyrim treatments at 5MRL showed a higher yellow component, giving brighter wines as a result (**right**).

As expected, the most important color changes in respect of the control wine were observed for iprovalicarb and mepanipyrim, especially at 5MRL (Table 2). In the color space defined by the International Commission on Illumination (CIELab), these wines showed a higher yellow component (b*) and a slight decrease of the red component (a*) (Figure 2). Consequently, an increment in the saturation (C_ab_*) was also observed. A significant increment of the yellow component was also confirmed by Glories with increments in the absorption at 420 nm, and as a result the color intensity and tonality increased. As stated above, these changes could be attributed to a higher polymerization rate observed for these wines that reduces the red component and increases the yellow color [36,37,38]. An important influence of mepanipyrim on the color of Tempranillo, Graciano and Mencía wines has also been observed in previous works [19,20].

Colorimetric differences can be considered as visually detectable when the value of the Euclidean distance (∆E_ab_*) is higher than 3.0 CIELab units [39]. As it can be seen in Table 2, values higher than 11 were obtained for iprovalicarb and mepanipyrim at 5MRL. Therefore, these wines could be perceived as different.

### 2.5. Antioxidant Activity

Fungicide residues in the must did not induce changes in the antioxidant activity of the wines in comparison to the control wine (11.54 mM·mL^−1^ of Trolox ± 0.32), even at the highest concentration assayed (5MRL). Mulero et al. [16] also observed that the antioxidant activity of Monastrell red wines was not altered by the presence of quinoxyfen, fluquinconazole, or famoxadone residues. However, the antioxidant activity found in those wines was lower than the one found in this study.

### 2.6. Relationship between Anthocyanins and Color for Differentiation among the Wines

**Figure 3:**
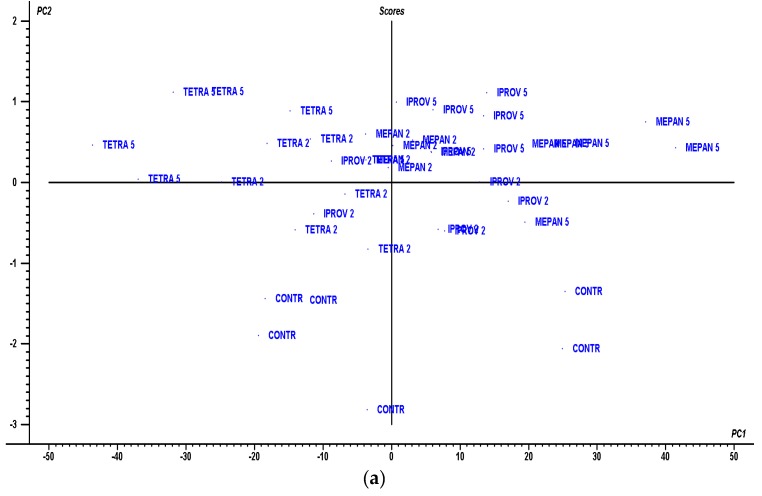

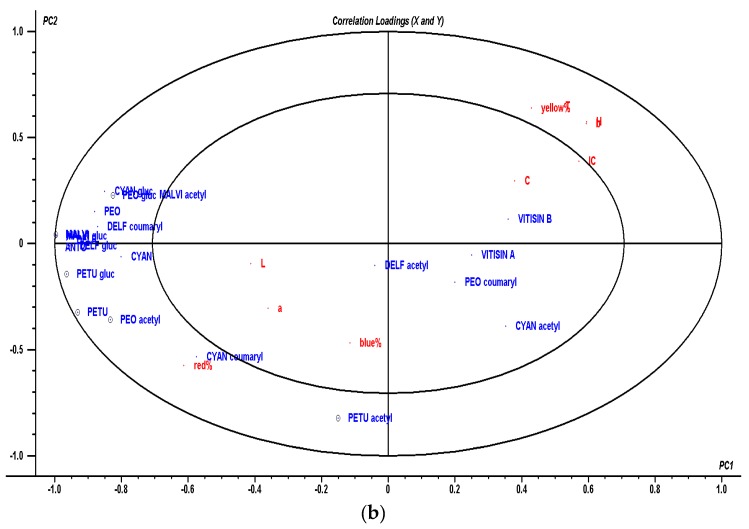
Two-dimensional PLS2: (**a**) scores plot for the control and treated wines, together with (**b**) correlations between the loadings of X (anthocyanin profiles in blue) and Y variables (color parameters in red).

**Figure 4:**
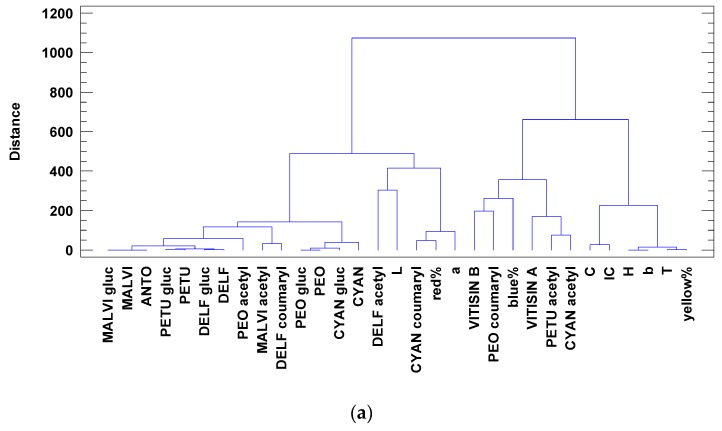

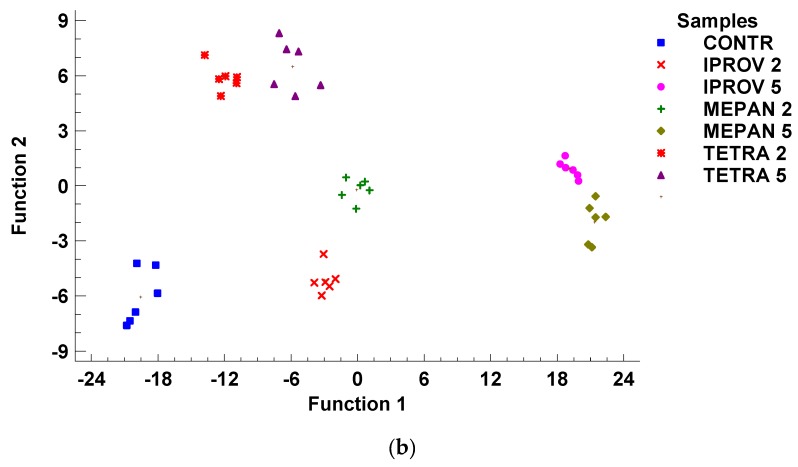
Dendrogram grouping variables according to the squared Euclidean similitude distance by Ward’s method (**a**). Four groups can be detected by cutting the dendrogram at a linking distance of about 450; from left to right, they are the same correlated variables shown in Figure 3b: upper-left, down-left, down-right, and upper-right quartiles. The upper-right quartile is the one with the color parameters more affected by iprovalicarb and mepanipyrim at 5MRL. The rest of the treated wines were more similar to the control wines. Discriminant biplot (**b**) for the classification variables of anthocyanin and color values.

With the aim of linking anthocyanin concentrations with color parameters, the following multivariate statistics were done. A PLS2 was used to correlate anthocyanin profiles with color data. As it can be seen in Figure 3, a two-factor model explaining 98% of the variance in X (anthocyanin profiles) and 71% of that in Y (color data) was obtained. The model was evaluated via the root mean square error for predictions (RMSEP), which was lower than 10 for Y values. The scores plot in Figure 3a shows how the target wines can be separated into different quadrants. PC2 can separate the control wines at the bottom from the rest. To separate the treated wines, PC1 is necessary; with its help, wines can be separated in the upper PC2 half in the following order from right to left: Mepan 5 > Iprov 5 > Mepan 2 = Iprov 2 = Tetra 2 > Tetra 5. All variables inside the ellipsoids between 0.7 and 1.0 for PCs 1 and 2 (Figure 3b) can be correlated among them (*r* > 0.700). Some color parameters such as hue angle (H), b*, IC, tonality, and yellow% were negatively correlated with mainly CYAN and PETU derivatives and red%. These correlations were connected to iprovalicarb and mepanipyrim treatments at 5MRL. The rest of the treated wines were more similar to the control wines. A cluster analysis of the anthocyanin concentrations and the color data (Figure 4a) allowed, by cutting the dendrogram at a linkage distance of 450, four main clusters or groups to be obtained for the same correlated variables shown in Figure 3b.

Since the anthocyanin profiles and color data can be used to separate wines, a discriminant analysis was performed (Figure 4b). Iprovalicarb and mepanipyrim treatments at 5MRL can be separated from the rest of the treatments using the linear discriminant function 1 (85% of variance), mainly because of the weight of the hue angle and b*. For the discrimination between iprovalicarb 5MRL and mepanipyrim 5MRL, the discriminant function 2 (9% of the variance) can be used, with the weights of cyanidin glucoside and delphinidin coumaryl being higher for iprovalicarb 5MRL.

## 3. Materials and Methods

### 3.1. Chemicals and Standards

The analytical standards of the fungicides iprovalicarb, mepanipyrim, and tetraconazole (Pestanal grade) were purchased from Supelco (Bellefonte, PA, USA). The standard of malvidin-3-*O*-glucoside chloride was purchased from Extrasynthese (Genay, Lyon, France). The solvents (residue analysis grade) were ethyl acetate, ultrapure water, and methanol from Sigma Aldrich (St. Louis, MO, USA) and ethanol from Scharlau (Barcelona, Spain). Trifluoroacetic acid was also purchased from Sigma Aldrich. Strata C18-E (2 g, 12 mL size) cartridges from Phenomenex (Torrance, CA, USA) were used for anthocyanins extraction.

### 3.2. Wine Samples

Red grapes, *Vitis vinifera* var. Monastrell from Jumilla (Murcia, Spain), were harvested in 2016. The grape characterization showed the following results: sugar content of 13.5%, pH 3.27, total acidity of 4.7 g·L^−1^, 2.41 g·L^−1^ of malic acid, and <0.01 g·L^−1^ of gluconic acid (determined using an Enological Multiparametric Analyzer Bacchus FTIR-Vis-UV MultiSpec (Tecnología Difusión Ibérica, Barcelona, Spain)). In addition, the amino acid content was determined through HPLC–DAD analysis after a sample derivatization with diethyl(ethoxymethylene) malonate, following the method described by Oliva et al. [40]. The total amino acid concentration in Monastrell grapes was 6.5 g·kg^−1^ and that of free amino acids lower than 4 g·kg^−1^, with glutamic acid (2.2 g·kg^−1^), proline (1.1 g·kg^−1^), and arginine (1.0 g·kg^−1^) being the main amino acids.

Vinifications were made, in triplicate, with 8 kg of destemmed and crushed grapes in the presence of fungicide residues: three with mepanipyrim, three with tetraconazole, and three with iprovalicarb standard solutions at two concentration levels corresponding to two and five times their MRL in grapes, established in the EU regulation [41,42,43,44], simulating critical agricultural practices. A control vinification (without fungicides) was also carried out, in triplicate, for comparative purposes. Twenty-one vinifications were carried out.

The winemaking process was performed as follows: crushed grapes (8 kg) were transferred to fermentation vessels (15 L) and sulphites (80 mg·kg^−1^) were added. The fermentation process was led in the presence of *Saccharomyces cerevisiae* var. *bayanus* Lalvin T73^™^ (25 g·HL^−1^) from Lallemand Wine (Montreal, QC, Canada). A maceration–fermentation step was done at 18 ± 2 °C to avoid volatile aroma compound losses. The temperature was controlled by recirculating refrigerated water around the fermentation vessel. During the maceration–fermentation time (10 days), a daily homogenization was performed to guarantee polyphenol extraction. Density and temperature were measured every day to control for delays or stoppages in the fermentation. The obtained must was pressed and left to ferment for another 4 days. After 7 days of sedimentation, the wine was transferred to other clean vessels and the lees were discarded. A clarification step was developed with bentonite (40 g·HL^−1^) and gelatin (8 g·HL^−1^) and, after 6 days, the wine was filtered (0.45 μm). The free SO_2_ content was analyzed in one sample of each experimental treatment obtaining values around 10 ± 1.5 mg·L^−1^, independently of the fungicide treatment applied. In order to stabilize the obtained wines, 30 mg·L^−1^ of SO_2_ was added to all the wines before bottling.

### 3.3. Fungicide Residue Analysis

Fungicides were extracted from the matrix following a QuEChERS multiresidue method that uses acetonitrile as an extraction solvent [45,46]. The obtained extract was acidified with formic acid and then directly injected into the liquid chromatograph. Liquid chromatography with tandem mass spectrometry (LC-MS/MS) analyses were performed following the chromatographic conditions described by Cermeño and coworkers [47].

### 3.4. Wine Characterization

#### 3.4.1. Enological Parameters

Alcoholic degree, total acidity, volatile acidity, pH, malic and lactic acid content, glucose/fructose ratio, dry extract, and total polyphenol index (TPI, based on the characteristic absorption at 280 nm of the benzene cycles of the majority of phenols [48]) were measured using an Enological Multiparametric Analyzer Bacchus FTIR-Vis-UV MultiSpec. The clarified wine samples were placed into the autosampler in 10 mL test tubes. Samples were suctioned and passed through an inert filter to prevent the entry of higher particles into the system (greater than 30 µm). A degasser prevented air or carbon dioxide from entering the measuring cells. Before the measurement, thermostatization of the sample at 25 °C took place. After measurement by the equipment, an automatic cleaning of the system took place. All wines were analyzed in duplicate. In order to be assured of a correct quantification through FTIR, wine control samples (whose enological parameters were previously characterized according to classical methods of the International Organization of Vine and Wine (OIV)) were incorporated into the sample sequence and also the influence of the target fungicides on the FTIR quantification was evaluated using hydro-alcoholic solutions (13%) of these fungicides.

#### 3.4.2. Color Determination

The chromatic characteristics were determined by means of a Beckman Coulter DU730 Life Science UV/Vis spectrophotometer (Fremont, CA, USA). After wine centrifugation (15 min at 1006× *g*) in a Rotina 35R centrifuge (Hettich Zentrifugen, Tuttlingen, Germany), spectrophotometric measures were taken using quartz cells from Hellma (Müllheim, Germany) of 1 mm and 1 cm of path length for undiluted and diluted samples, respectively. The visible spectrum (200–800 nm) was recorded (Δλ = 5 nm) to calculate the colorimetric indices (% yellow, % red, % blue, tonality, and color intensity) according to Glories [49]. All measurements were carried out in triplicate. A hydro-alcoholic solution (12% ethanol) was used as a blank. CIELab parameters (lightness (L*), color components (a* and b*), Chroma (C_ab_*), and hue angle (h_ab_)) were also determined [50].

#### 3.4.3. Phenolic Composition and Distribution

Both the total anthocyanin content and its distribution into monomeric, polymeric, and copigmented fractions were determined according to Boulton [51]. The monomeric anthocyanin profile was determined according to Briz-Cid et al. [17]. All the anthocyanins (malvidin, petunidin, peonidin, delphinidin, and cyanidin derivatives) were quantified as malvidin-3-*O*-glucoside by a standard calibration curve, with adsorption being measured at 520 nm.

#### 3.4.4. Antioxidant Activity

The samples were analyzed following the method reported by Brand-Williams et al. [52]. The scavenging activity of the free radicals, using the α,α-diphenyl-β-picrylhydrazyl (DPPH) free radical reaction, was evaluated. The results were expressed as equivalent mM·mL^−1^ of Trolox^®^ (6-hydroxy-2,5,7,8-tetramethylchroman-2-carboxylic acid), a vitamin E analog [16].

### 3.5. Multivariate Statistical Analysis

Partial Least Squares Regression (PLS2) analysis was used for relating CIELab color parameters (Y matrix) and anthocyanin concentrations (X matrix), through a linear multivariate model, performed with The Unscrambler software from CAMO. The root mean square error for predictions (RMSEP) was used to validate the obtained model. The RMSEP calculates the residual variation as a function of the number of components. The result presents, for each component, the estimated cross validation error and the cumulative percentage of variance explained. A cluster analysis to group variables (color parameters and phenolic concentrations) based on the squared Euclidean similitude distance by Ward’s method [53] was performed, together with discriminant analyses to separate the wines, using the statistical software package Statgraphics Centurion XVI from StatPoint Technologies Inc.

## 4. Conclusions

High fungicide dissipation rates along the winemaking process were obtained for mepanipyrim and tetraconazole (higher than 91%) while around 27% of the iprovalicarb concentration added to the must remained in the final wine. For the first time, the effect of these fungicides on the color and phenolic profile of Monastrell wines was evaluated. While no significant changes were observed for tetraconazole, those wines obtained in the presence of mepanipyrim and iprovalicarb residues, especially at the highest concentration assayed (5MRL), were different from the control wine. For both mepanipyrim and iprovalicarb, an increase of the volatile acidity, the lactic acid content, the total phenolic index, and the percentage of polymeric anthocyanins was observed, while a decrease of the total monomeric anthocyanins was found. The color of these wines was also different (higher yellow component, color intensity, tonality, and saturation) in comparison with the control wines. Since vinifications were done at the same time following the same enological conditions and also compared with a control vinification (without fungicides), the behavior observed should be attributed only to the presence of fungicide residues in the must that affected the yeast development and allowed a rapid development of bacteria. This is also in concordance with the high concentration of both fungicides in the must after pressing, in comparison to tetraconazole.

In conclusion, residues of the considered fungicides in wine, beyond the related possible health risk, can modify the fermentation process, the wine microbiome, and, consequently, the physicochemical properties and the quality of the wine. Although, the major effects on wine quality were observed for iprovalicarb and mepanipyrim, it is important to remark that antibotritics (such as mepanipyrim) are the last fungicide treatment applied on vineyards (about one month before harvest). Therefore, they are likely to be present at higher concentration levels. In any case, the effect observed on wine quality is fungicide concentration dependent. Therefore, under good agricultural practices, respecting the MRLs on grapes, the potential individual effects of those fungicides will be lower than those observed in this work.

## Figures and Tables

**Table 1:** Fungicide concentrations (mg·kg^−1^) found in the different liquid and solid phases separated along the winemaking process (average ± standard deviation) with the final dissipation (% in mass units) determined in the clarified wine. In addition, the mean transfer factor from grapes to wine and the acceptable daily intake (ADI, %) are shown.

Step	Iprovalicarb		Mepanipyrim		Tetraconazole	
	10 mg·kg^−1^ (5MRL)	4 mg·kg^−1^ (2MRL)	10 mg·kg^−1^ (5MRL)	4 mg·kg^−1^ (2MRL)	2.5 mg·kg^−1^ (5MRL)	1 mg·kg^−1^ (2MRL)
Must-wine after pressing (5.3 L)	6.44 ± 0.21	3.19 ± 0.08	1.79 ± 0.04	0.68 ± 0.03	0.92 ± 0.18	0.37 ± 0.03
Grape pomace (2.2 kg)	12.12 ± 2.39	5.56 ± 0.08	12.41 ± 1.40	5.04 ± 0.28	5.90 ± 0.45	2.63 ± 0.46
Wine (3.5 L)	6.23 ± 0.31	2.86 ± 0.43	0.87 ± 0.05	0.33 ± 0.01	0.46 ± 0.04	0.20 ± 0.01
Lees (0.5 kg)	9.26 ± 1.56	3.17 ± 0.03	3.22 ± 0.08	1.34 ± 0.06	1.59 ± 0.14	0.78 ± 0.09
Clarified wine (3.5 L)	5.99 ± 0.13	2.60 ± 0.05	0.68 ± 0.04	0.25 ± 0.02	0.47 ± 0.04	0.21 ± 0.03
ADI (%)	85.6	37.1	12.1	4.5	25.2	11.3
Mass transfer factor	0.60	0.65	0.07	0.06	0.19	0.21
Fungicide dissipation (%)	71.6	73.8	97.3	97.0	90.8	91.8

**Table 2:** Enological and color parameters of the obtained wines in the presence and absence of fungicides (average ± standard deviation).

	CONTROL	IPROVALICARB 2MRL	IPROVALICARB 5MRL	MEPANIPYRIM 2MRL	MEPANIPYRIM 5MRL	TETRACONAZOLE 2MRL	TETRACONAZOLE 5MRL
Enological parameters							
Alcoholic degree (% vol)	13.73 ^a^ ± 0.63	13.95 ^ab^ ± 0.07	13.83 ^a^ ± 0.05	14.03 ^ab^ ± 0.03	14.04 ^ab^ ± 0.03	14.27 ^b^ ± 0.07	14.34 ^b^ ± 0.04
Acidity (g/L tartaric acid)	6.17 ^a^ ± 0.22	6.48 ^b^ ± 0.06	6.85 ^c^ ± 0.05	6.61 ^b^ ± 0.08	6.53 ^b^ ± 0.07	6.15 ^a^ ± 0.03	6.18 ^a^ ± 0.05
Volatile acidity (g/L acetic acid)	0.42 ^a^ ± 0.05	1.66 ^d^ ± 0.03	3.62 ^e^ ± 0.06	1.67 ^d^ ± 0.04	3.63 ^e^ ± 0.14	0.66 ^b^ ± 0.02	1.10 ^c^ ± 0.01
pH	3.43 ^a^ ± 0.02	3.46 ^b^ ± 0.01	3.49 ^c^ ± 0.01	3.45 ^b^ ± 0.01	3.46 ^b^ ± 0.01	3.42 ^a^ ± 0.01	3.45 ^b^ ± 0.01
Malic acid (g/L)	1.96 ^d^ ± 0.16	0.00 ^a^ ± 0.00	0.00 ^a^ ± 0.00	0.08 ^a^ ± 0.09	0.00 ^a^ ± 0.00	1.57 ^c^ ± 0.06	0.85 ^b^ ± 0.10
Lactic acid (g/L)	0.34 ^a^ ± 0.04	3.03 ^d^ ± 0.10	6.98 ^e^ ± 0.18	2.93 ^d^ ± 0.15	6.91 ^e^ ± 0.23	0.79 ^b^ ± 0.03	1.90 ^c^ ± 0.08
Glucose/fructose ratio	0.18 ^a^ ± 0.16	0.00 ^b^ ± 0.00	0.00 ^b^ ± 0.00	0.00 ^b^ ± 0.00	0.00 ^b^ ± 0.00	0.01 ^b^ ± 0.01	0.00 ^b^ ± 0.00
Dry extract (g/L)	24.05 ^a^ ± 0.58	27.45 ^c^ ± 0.35	32.70 ^d^ ± 0.54	27.07 ^bc^ ± 0.55	33.52 ^e^ ± 0.29	24.55 ^a^ ± 0.37	26.30 ^b^ ± 0.21
Total Phenol Index (TPI)	44.29 ^a^ ± 0.57	45.48 ^ab^ ± 0.80	47.36 ^cd^ ± 0.48	45.73 ^b^ ± 0.97	48.33 ^d^ ± 0.24	44.37 ^a^ ± 0.66	46.41 ^bc^ ± 0.94
Colorimetric indexes							
% yellow	32.48 ^a^ ± 0.25	32.92 ^b^ ± 0.31	34.25 ^c^ ± 0.06	33.26 ^b^ ± 0.23	34.20 ^c^ ± 0.19	32.94 ^b^ ± 0.15	33.15 ^b^ ± 0.16
% red	54.13 ^d^ ± 0.24	53.73 ^c^ ± 0.28	53.23 ^a^ ± 0.13	53.65 ^bc^ ± 0.19	53.32 ^ab^ ± 0.08	53.90 ^cd^ ± 0.23	54.02 ^cd^ ± 0.27
% blue	13.39 ^c^ ± 0.39	13.35 ^c^ ± 0.48	12.52 ^a^ ± 0.11	13.10 ^bc^ ± 0.08	12.47 ^a^ ± 0.12	13.16 ^bc^ ± 0.16	12.83 ^ab^ ± 0.26
Tonality	60.00 ^a^ ± 0.46	61.27 ^bc^ ± 0.57	64.34 ^d^ ± 0.24	61.99 ^c^ ± 0.63	64.14 ^d^ ± 0.44	61.10 ^b^ ± 0.50	61.36 ^bc^ ± 0.50
Color intensity	12.09 ^ab^ ± 0.22	12.90 ^cd^ ± 0.46	12.98 ^cd^ ± 0.37	12.80 ^c^ ± 0.64	13.46 ^d^ ± 0.23	11.87 ^a^ ± 0.18	12.54 ^bc^ ± 0.20
CIELab space							
a*	50.46 ^b^ ± 0.17	50.24 ^b^ ± 0.38	49.22 ^a^ ± 0.62	50.19 ^ab^ ± 0.98	49.98 ^ab^ ± 0.27	49.91 ^ab^ ± 0.42	50.84 ^b^ ± 0.64
b*	5.33 ^a^ ± 0.36	9.18 ^bc^ ± 0.59	16.34 ^d^ ± 0.82	9.73 ^c^ ± 0.78	17.21 ^d^ ± 0.18	5.72 ^a^ ± 0.29	8.47 ^b^ ± 0.90
L*	45.97 ^ab^ ± 0.79	44.53 ^a^ ± 1.08	45.78 ^ab^ ± 0.78	45.03 ^a^ ± 1.59	44.77 ^a^ ± 0.67	46.90 ^b^ ± 0.52	45.65 ^ab^ ± 0.33
C_ab_*	50.68 ^ab^ ± 0.22	51.07 ^abc^ ± 0.43	51.87 ^cd^ ± 0.84	51.13 ^abc^ ± 1.10	52.86 ^d^ ± 0.23	50.24 ^a^ ± 0.40	51.55 ^bc^ ± 0.70
h_ab_	6.03 ^a^ ± 0.40	10.35 ^bc^ ± 0.62	18.36 ^d^ ± 0.67	10.96 ^c^ ± 0.68	19.00 ^d^ ± 0.24	6.54 ^a^ ± 0.35	9.45 ^b^ ± 0.96
∆E_ab_*		4.12	11.08	4.50	11.95	1.15	3.18

^a, b, c, d, e^: statistical differences according to the ANOVA test (*p* < 0.05) between control and treated wines.

## Supplementary Materials

Supplementary materials can be found at https://www.mdpi.com/1422-0067/20/6/1447/s1.
