# Supplementary material for: Dissipation of Three Fungicides and Their Effects on Anthocyanins and Color of Monastrell Red Wines
Source: Int J Mol Sci. 2019 Mar 22;20(6):1447. doi: 10.3390/ijms20061447 (PMC6470954; doi:10.3390/ijms20061447)
Supplement: Supplementary file 1 [file ijms-20-01447-s001.pdf]

Table S1. Mean values ± standard deviation (n=3) of the monomeric anthocyanins content for the control and the treated wines.

Wines	CONTROL	IPROVALICARB 2MRL	IPROVALICARB 5MRL	MEPANIPYRIM 2MRL	MEPANIPYRIM 5MRL	TETRACONAZOLE 2MRL	TETRACONAZOLE 5MRL
**Anthocyanins by UV/Vis**							
Monomeric (%)	41.52^{d} ± 1.89	27.76^{b} ± 2.83	13.41^{a} ± 1.45	24.90^{b} ± 0.79	10.72^{a} ± 1.18	39.30^{a} ± 1.14	34.02^{c} ± 1.43
Copigmented (%)	15.14^{a} ± 2.08	14.17^{a} ± 1.40	15.52^{a} ± 2.17	18.80^{b} ± 0.81	14.76^{ab} ± 2.55	15.26^{a} ± 1.86	14.66^{a} ± 1.62
Polymeric (%)	42.22^{a} ± 2.45	56.80^{d} ± 0.70	71.06^{e} ± 0.83	56.30^{d} ± 1.06	72.82^{e} ± 0.48	45.44^{b} ± 0.83	51.32^{c} ± 0.99
**TOTAL Anthocyanins (absorbance units)**	6.36^{ab} ± 0.12	6.71^{bc} ± 0.11	6.86^{cd} ± 0.21	6.73^{c} ± 0.37	7.09^{d} ± 0.16	6.23^{a} ± 0.11	6.66^{bc} ± 0.16
**Monomeric anthocyanins by HPLC**							
**Malvidin derivatives**							
malvidin-3-*O*-glucoside	60.20^{cde} ± 4.19	51.61^{bc} ± 5.41	50.14^{b} ± 2.56	54.35^{bcd} ± 2.12	40.56^{a} ± 3.89	60.53^{de} ± 4.10	66.34^{e} ± 6.38
malvidin-3-*O*-(6-*O*-acetyl)glucoside	0.87^{ab} ± 0.06	0.78^{a} ± 0.09	0.85^{ab} ± 0.04	0.83^{ab} ± 0.06	0.81^{ab} ± 0.08	0.87^{ab} ± 0.08	0.94^{a} ± 0.12
vitisin A	2.39^{ab} ± 0.17	2.28^{ab} ± 0.14	2.32^{ab} ± 0.22	2.25^{ab} ± 0.21	2.39^{b} ± 0.34	1.98^{a} ± 0.09	2.13^{ab} ± 0.11
vitisin B	1.92^{a} ± 0.12	2.63^{b} ± 0.30	2.48^{b} ± 0.11	2.33^{ab} ± 0.17	2.64^{b} ± 0.39	2.26^{ab} ± 0.10	2.45^{b} ± 0.26
**subTOTAL (mg L^{-1}) (%)**	**65.38^{cde} ± 3.99**	**57.30^{bc} ± 5.30**	**55.80^{b} ± 2.57**	**59.75^{bcd} ± 2.10**	**46.40^{a} ± 4.34**	**65.64^{de} ± 4.11**	**71.85^{e} ± 6.73**
**Petunidin derivatives**							
petunidin-3-*O*-glucoside	13.94^{cd} ± 0.49	11.38^{ab} ± 1.25	10.64^{ab} ± 0.72	11.88^{bc} ± 0.40	9.47^{a} ± 1.46	13.86^{cd} ± 0.56	14.92^{d} ± 1.89
petunidin-3-*O*-(6-*O*-acetyl)glucoside	2.53^{c} ± 0.35	1.98^{b} ± 0.28	1.19^{a} ± 0.05	1.16^{a} ± 0.03	1.15^{a} ± 0.20	1.19^{a} ± 0.03	1.17^{a} ± 0.03
**subTOTAL (mg L^{-1}) (%)**	**16.46^{d} ± 0.56**	**13.36^{bc} ± 1.52**	**11.83^{ab} ± 0.71**	**13.05^{bc} ± 0.43**	**10.61^{a} ± 1.75**	**15.05^{cd} ± 0.58**	**16.09^{d} ± 1.91**
**Delphinidin derivatives**							
delphinidin-3-*O*-glucoside	7.50^{cd} ± 0.19	6.66^{bc} ± 0.71	6.04^{ab} ± 0.34	6.86^{bcd} ± 0.32	5.46^{a} ± 0.82	7.88^{de} ± 0.31	8.82^{e} ± 0.93
delphinidin-3-*O*-(6-*O*-p-coumaroyl)glucoside	3.89^{bc} ± 0.53	3.51^{abc} ± 0.69	3.36^{abc} ± 0.26	3.28^{ab} ± 0.36	2.82^{a} ± 0.35	3.66^{abc} ± 0.51	4.24^{c} ± 0.68
delphinidin-3-*O*-(6-*O*-acetyl)glucoside	0.94^{a} ± 0.03	0.83^{a} ± 0.05	0.91^{a} ± 0.04	0.82^{a} ± 0.07	0.91^{a} ± 0.13	0.90^{a} ± 0.08	0.92^{a} ± 0.04
**subTOTAL (mg L^{-1}) (%)**	**12.34^{bcd} ± 0.72**	**11.00^{abc} ± 1.33**	**10.31^{ab} ± 0.60**	**10.95^{abc} ± 0.42**	**9.20^{a} ± 1026**	**12.34^{cd} ± 0.68**	**13.98^{d} ± 1.53**
**Peonidin derivatives**							
peonidin-3-*O*-glucoside	5.40^{ab} ± 0.26	5.59^{ab} ± 0.65	5.34^{ab} ± 0.21	5.57^{ab} ± 0.27	4.84^{a} ± 0.74	6.01^{bc} ± 0.34	6.63^{c} ± 0.63
peonidin-3-*O*-(6-*O*-acetyl)glucoside	0.70^{c} ± 0.09	0.42^{b} ± 0.06	0.41^{b} ± 0.03	0.42^{b} ± 0.02	0.30^{a} ± 0.02	0.60^{c} ± 0.05	0.60^{c} ± 0.06
peonidin-3-*O*-(6-*O*-p-coumaroyl)glucoside	0.05^{ab} ±≤ 0.01	0.05^{ab} ± 0.01	0.05^{b} ±≤ 0.01	0.05^{ab} ±≤ 0.01	0.05^{ab} ±≤ 0.01	0.05^{ab} ±≤ 0.01	0.04^{a} ±≤ 0.01
**subTOTAL (mg L^{-1}) (%)**	**6.15^{ab} ± 0.27**	**6.06^{ab} ± 0.69**	**5.80^{ab} ± 0.23**	**6.03^{ab} ± 0.26**	**5.19^{a} ± 0.74**	**6.66^{bc} ± 0.35**	**7.27^{c} ± 0.68**
**Cyanidin derivatives**							
cyanidin-3-*O*-glucoside	1.50^{ab} ± 0.02	1.51^{ab} ± 0.15	1.49^{ab} ± 0.06	1.60^{b} ± 0.07	1.29^{a} ± 0.15	1.85^{c} ± 0.09	2.12^{d} ± 0.19
cyanidin-3-*O*-(6-*O*-acetyl)glucoside	0.74^{cd} ± 0.05	0.84^{d} ± 0.12	0.69^{bc} ± 0.09	0.69^{bc} ± 0.03	0.57^{ab} ± 0.04	0.48^{a} ± 0.06	0.53^{a} ± 0.04
cyanidin-3-*O*-(6-*O*-p-coumaroyl)glucoside	0.54^{c} ± 0.06	0.46^{bc} ± 0.04	0.41^{ab} ± 0.02	0.45^{abc} ± 0.02	0.38^{a} ± 0.04	0.49^{c} ± 0.03	0.53^{c} ± 0.06
**subTOTAL (mg L^{-1}) (%)**	**2.78^{b} ± 0.06**	**2.80^{b} ± 0.22**	**2.59^{b} ± 0.11**	**2.74^{b}** ± 0.08	**2.25^{a} ± 0.14**	**2.82^{b} ± 0.12**	**3.17^{c} ± 0.25**
**TOTAL monomeric anthocyanins (mg L^{-1})**	**103.11^{cd} ± 4.70**	**90.52^{bc} ± 8.57**	**86.33^{b} ± 4.01**	**92.53^{bc} ± 2.47**	**73.64^{a} ± 7.58**	**102.60^{cd} ± 5.22**	**112.36^{d} ± 10.87**

^{a,b,c,d,e}: statistical differences according to the *ANOVA* test ($p < 0.05$)
